# Shuyu Capsules Relieve Premenstrual Syndrome Depression by Reducing 5-HT_3A_R and 5-HT_3B_R Expression in the Rat Brain

**DOI:** 10.1155/2016/7950781

**Published:** 2016-09-20

**Authors:** Fang Li, Jizhen Feng, Dongmei Gao, Jieqiong Wang, Chunhong Song, Sheng Wei, Mingqi Qiao

**Affiliations:** ^1^Fengtai Maternal and Children's Health Hospital of Beijing, Beijing 100069, China; ^2^Department of Radiology, Shandong Provincial Hospital Affiliated to Shandong University, Jinan 250021, China; ^3^Key Laboratory for Classical Theory of Traditional Chinese Medicine of Education Ministry, Shandong University of Traditional Chinese Medicine, Jinan 250355, China

## Abstract

The effects of the Shuyu capsule on 5-HT_3A_R and 5-HT_3B_R expression in a rat model of premenstrual syndrome (PMS) depression and on 5-HT_3A_R and 5-HT_3B_R expression and hippocampal neuron 5-HT_3_ channel current were investigated, to elucidate its mechanism of action against PMS depression. PMS depression model rats were divided into depression and Shuyu- and fluoxetine-treated groups, which were compared to control rats for frontal lobe and hippocampal 5-HT_3A_R and 5-HT_3B_R expression and behavior. The depressed model rats displayed symptoms of depression, which were reduced in treated and normal control rats. Frontal lobe and hippocampal 5-HT_3A_R and 5-HT_3B_R levels were significantly higher in the model versus the control group and were significantly lower in the Shuyu group. As compared to control rats, the 5-HT_3_R channel current in the model group was significantly higher; the 5-HT_3_R channel current in hippocampal neurons treated with serum from Shuyu group rats was significantly lower than that in those treated with model group serum. Thus, PMS depression may be related to 5-HT_3A_R and 5-HT_3B_R expression and increased 5-HT_3_ channel current. Shuyu capsules rectified abnormal 5-HT_3A_R and 5-HT_3B_R expression and 5-HT_3_ channel current changes in a rat model; this finding may provide insight into treating PMS depression.

## 1. Introduction

Premenstrual syndrome (PMS), a disease commonly encountered in clinical gynecology, refers to a series of moods, mental and physical symptoms, and signs occurring in the premenstrual period (luteal phase) of reproductive-aged women, including irritability, anxiety, nervousness, breast distention pain, and headache. The abovementioned symptoms automatically mitigate or disappear after menstruation but recur regularly with each menstrual cycle [1, 2]. Epidemiological surveys conducted worldwide have revealed that reproductive-aged women undergo one or more mood and physical PMS-related symptoms [3] that significantly influence their physical and mental health, as well as their quality of life. Moreover, the incidence of PMS has been increasing annually, attracting increased attention from the medical field.

PMS depression is a major type of PMS with features of depression, sullenness, chest distress, sighing, and a depressed mood [4, 5]. It has been reported that monoamine neurotransmitters including 5-hydroxytryptamine 1A receptor, 5-hydroxytryptamine 2A receptor, 5-hydroxytryptamine 2C receptor, and 5-hydroxytryptamine 3 receptor are responsible for premenstrual syndromes [6–11]. Widely distributed in the central nervous system and peripheral nervous system, 5-HT_3_R influences the metabolism of neurotransmitters in brain tissues (such as 5-HT, DA, CKK, and GABA) and then influences receptor proteins related to mood, memory, and mental health conditions. To date, five subtypes of the 5-HT_3_R, namely, the 5-HT_3A_R, 5-HT_3B_R, 5-HT_3C_R, 5-HT_3D_R, and 5-HT_3E_R, have been discovered. Of these, the first two are the major subtypes. In the central nervous system, 5-HT_3_ receptor subtypes are involved in the pathological processes of depression [12, 13], anxiety, and withdrawal symptoms, and 5-HT_3_R is involved in resistance to depression and anxiety. A previous study has indicated that 5-HT_3_R antagonists can enhance the antidepressant effect of 5-HT reuptake inhibitors [14].

Shuyu capsules, a commercially available herbal prescription of traditional Chinese medicine (TCM), is composed of four herbal ingredients: Radix Bupleuri (*Bupleurum chinense* DC.), Radix Paeoniae Alba (*Paeonia lactiflora* Pall.), Rhizoma Cyperi (*Cyperus rotundus* Linn.), and Radix Glycyrrhizae (*Glycyrrhiza uralensis* Fisch.). It has been confirmed that it mitigates the expression of PMS depression symptoms in patients in clinical experiments. Animal experiments have also confirmed that improvements related to such symptoms are concentrated in the relevant cerebral areas. It is often technologically difficult to identify and purify active constituents in TCM prescriptions, and thus they are often not recognized by doctors of western medicine or scientists. We have focused on testing the curative effects of TCM using modern clinical experiments to evaluate the effect of TCM prescriptions [15, 16], with the intent of retaining effective TCM treatments and abandoning ineffective ones. In this respect, a previous study has demonstrated that Shuyu capsules, confirmed to mitigate PMS depression (number 2008L11169), mainly target 5-HT_3B_R expression levels in areas of the hippocampus and hypothalamus [17, 18]. Nevertheless, the mechanisms by which the Shuyu capsule exerts its functions in these cerebral areas require further explanation.

In the present study, the Shuyu capsule, which comprises a mixture of natural medicinal materials, was used without consideration of the unknown interactions of these compounds [19]. An animal model of PMS depression, created using a chronic restraint-stress method, was given either Shuyu capsules or fluoxetine, and the distribution and protein expression of 5-HT_3A_R and 5-HT_3B_R in the prefrontal cortex and hippocampus were determined and compared. Additionally, a drug-containing serum derived from these animals was used as a method of drug delivery in a neuronal culture system, and the expression of 5-HT_3_R protein and changes in the 5-HT_3_ channel current were investigated to determine the relationship between 5-HT_3_R expression and PMS depression at the cellular and molecular levels. In this way, we confirmed the target cerebral areas of the Shuyu capsule in regulating PMS depression and gained insight into its mechanism of action.

## 2. Materials and Methods

### 2.1. Laboratory Animals and Ethics Statement

Healthy female SPF Wistar rats weighing 160–180 g were selected. Animals had free access to water and food. The feeding room temperature was 24 ± 1°C and the relative humidity was 50 ± 10%. Animals were provided by the Laboratory Animal Center of Shandong Traditional Chinese Medicine University, license number SCXK (LU) 2011-0003. In the following assays, 72 rats were randomized into 4 groups (*n* = 18 for behavioral assays; *n* = 6 for immunohistochemistry; *n* = 6 for western blotting; *n* = 6 for serum collection): control group, model group, Shuyu administration group, and fluoxetine administration group. Rats in control group did not give any stimulation while model group were simulated with leg bounded. As to Shuyu capsule and fluoxetine administration groups, medicines were chronically administered to rats when modeling was at the same time.

Laboratory animals were provided care according to “*The Care and Use of Laboratory Animals*” by the Laboratory Animal Center of Shandong University of Traditional Chinese Medicine.

### 2.2. Chemicals and Drugs

The Shuyu capsule (clinical approval number 2008L11169) used in this experiment was composed of Radix Paeoniae Alba, Radix Bupleuri, Rhizoma Cyperi, and liquorice. Fluoxetine was used as the positive control (Eli Lilly Co., Indianapolis, IN, USA; approval number H20090463). Both Shuyu capsule (0.41 g/Kg/d) and fluoxetine (10 mg/Kg/d) were intragastrically administered to animals for 5 days when experiment needed so.

### 2.3. Primary Culture of Hippocampal Neurons

The pregnant rats used here were not abovementioned grouped rats and the primary culture steps were as follows. CO_2_ was used to anesthetize the rats; their heads were removed rapidly. Alcohol was sprayed on the abdomens of the pregnant rats, the abdomens were split and the embryo was stripped out. Embryo head was removed after stripping and placed into iced phosphate-buffered saline (PBS). The skull was split to expose and open the cerebral cortex. After locating the hippocampus, it was removed and placed into 10 mL of PBS in a centrifugal tube containing ice. Trypsin was added, and the specimens were incubated at 37°C for 20 min; tubes were shaken every 5 min. Cells were collected from the trypsinized samples and washed in DMEM 3 times, and 4 h later, the medium was changed to NBG medium (Neurobasal : B27 : L-Glutamine = 100 : 2 : 1, Gibco/Life Technologies, Carlsbad, CA, USA) for incubation.

### 2.4. Determination of the Estrous Cycle

All grouped rats were weighed, recorded, and marked with picric acid, and their estrous cycle was determined using the vaginal smear microscopic examination method [20, 21]. On a proestrus vaginal smear, epithelial cell nuclei and a few keratinocytes are present, while on an estrum vaginal smear, anucleate keratinocytes and a few epithelial cells are present. On a postestrus vaginal smear, leukocytes, keratinocytes, and epithelial cell nuclei are found, and on an anestrus vaginal smear, there are large numbers of leukocytes and few epithelial cells and myxocytes.

### 2.5. Generation of a PMS Depression Rat Model

Rats tend to be active at the acceptance stage (proestrus and estrus), and their estrous behaviors abate or even disappear at the nonacceptance stage (postestrus and anestrus). Rats with regular estrus behaviors and at the nonacceptance stage, and which obtained similar scores in the open-field test and sucrose-preference test, were selected for model building.

The model was generated as previously reported, with some modifications [17], and the specific steps were as follows. The four legs of the rats were bound crosswise; that is, the front leg and hind leg on the opposite side were bound with sterile gauze (width: 2 cm, and of appropriate length) so as to prevent them from moving freely. The rats were bound in such a way that they were able to move slightly and obtain some food, and the modeling lasted for 5 days. During this process, the same dosage of sterile drinking water was provided to the control group.

### 2.6. Behavioral Assays

The open-field test [22, 23] was used to measure the locomotor activity of the rats. We employed the XR-Xvideo (including the XR-Xvideo animal behavior video analytical system) for this test. Under dim red light, experimenters held the distal third of the rats' tails and placed them at the center of an open-field test box (size: 50 cm × 50 cm × 40 cm) with black walls and a black floor. The video system recorded behavioral changes during a 5 min period, and the software automatically recorded the general path of their movement. The model test was conducted before experimental animal screening and after model building and drug administration.

Furthermore, the sucrose-preference experiment [24] was used to measure the level of reward response in the rats [25]. Depressive animals showed a general decline in sucrose preference, representing an anhedonia symptom. In the experiment, two bottles of water were provided to the rats for their free selection over a 24 h period. One bottle contained a 1% sucrose aqueous solution, and the other contained pure water. The two bottles were located on opposite sides of the cage. Before the experiment, the rats had free access to water and food. The consumption of tap water and sucrose water was measured by weighing the bottles. The sucrose preference was expressed as the percentage of consumed sucrose water to the total liquid consumed. Sucrose-preference rate = sucrose water consumption (g)/(sucrose water consumed (g) + water consumption (g)) × 100%.

### 2.7. Immunohistochemistry

According to a stereotaxic atlas of rats, 3 mm of the anterior brain, containing the frontal lobe, and 5 mm of the middle section, containing the hippocampus and hypothalamus, were removed. The removed cerebral tissues were fixed in triformol (Sigma-Aldrich, St. Louis, MO, USA) for 1 week at a temperature of 4°C. Samples were dehydrated in a gradient series of hydrous ethanol and were then treated with xylene to make the samples transparent. Dehydrated samples of cerebral tissues were embedded in paraffin and then sliced by microtome (RM2015; Leica, Wetzlar, Germany) at a thickness of 5 *μ*m. After deparaffinizing and hydration treatments, the sections were again washed with PBS three times, before being incubated with primary antibodies overnight at a temperature of 4°C. The primary antibodies included goat polyclonal antibodies to the 5-HT_3A_ receptor (Abcam, Cambridge, MA, USA; ab51950, 1 : 500) and to the 5-HT_3B_ receptor (Abcam; ab115023, 1 : 50). After washing, the sections were incubated with secondary antibodies (Cy3-labeled donkey anti-goat IgG (H+L); Beyotime, Haimen, China; A0505, 1 : 2000; and Alexa Fluor 488-labeled goat anti-rabbit IgG (H+L); Beyotime, A0423, 1 : 2000) for 2 h. A laser scanning confocal microscope (ZEISS LSM710) was then used to observe the samples [26, 27].

### 2.8. Western Blot

The primary antibodies used for western blotting included a goat polyclonal to the 5-HT_3A_ receptor (Abcam, ab51950, 1 : 200), a goat polyclonal to the 5-HT_3B_ receptor (Santa Cruz, Dallas, TX, USA; Sc-51198, 1 : 200), and a mouse monoclonal to *β*-actin (Sigma, A1978, 1 : 1000). The secondary antibodies included rabbit anti-goat IgG-HRP (Abcam, ab6741, 1 : 2000), donkey anti-goat IgG-HRP (Santa Cruz, Sc-2020, 1 : 1000), and goat anti-mouse IgG-HRP (Santa Cruz, Sc-2005, 1 : 2000). Results were monitored by chemiluminescence. A GE Healthcare LAS4000 (Little Chalfont, UK) was used to collect signals, and the built-in IQTL analytic software was adopted for the statistical analysis.

### 2.9. Serum Collection

About 90 min after last administration of Shuyu capsule/fluoxetine, rats received intraperitoneal injection of 1% pentobarbital sodium (CAS: 57-33-0, Sigma, USA). Then, blood was collected via aorta abdominalis. Blood samples were centrifuged (3000 rpm for 20 min), and the serum was separated and stored at −80°C.

### 2.10. Whole-Cell Patch Clamp

The voltage clamp mode of the Axon MultiClamp 700B (Molecular Devices, Sunnyvale, CA, USA) was used in this experiment [28]. The clamp voltage was −70 mV, the frequency of the Bessel filter was 2.9 kHz, and the sampling frequency was 20 kHz. The microelectrode was drawn using a microelectrode puller (P97, Sutter, Sacramento, CA, USA) with a resistance of 3–5 MΩ. The microelectrode manipulator (M200, Sutter) of an inverted microscope (IX71, Olympus, Tokyo, Japan) was used to control the electrode entering solutions. When the resistance stabilized, a negative pressure injector was used to break the patch to perform a whole-cell recording mode. Prior to or after breaking the patch, capacitance compensations were made without series-resistance compensation. After setting up the whole-cell mode, cells with series resistance Ra < 20 MΩ were included in the experiment. Phenylbiguanide (PBG; 10 *μ*m) drug administration resulted in quick perfusion of the Y tube beside cells. All experiments were conducted at an indoor temperature of 21 ± 1°C.

### 2.11. Statistical Analysis

Two-way ANOVA was selected for the open-field test and the sucrose-preference test; GraphPad Prism 5 was used for the one-way ANOVA test. PClamp 10.0 was used for the whole-cell patch clamp recording of the 5-HT_3_R current. All data are shown as the mean ± SEM, with the significance level set at *P* < 0.05.

## 3. Results

### 3.1. Shuyu Capsules Can Effectively Mitigate Depressive Behavior in a Rat PMS Model

**Figure 1:**
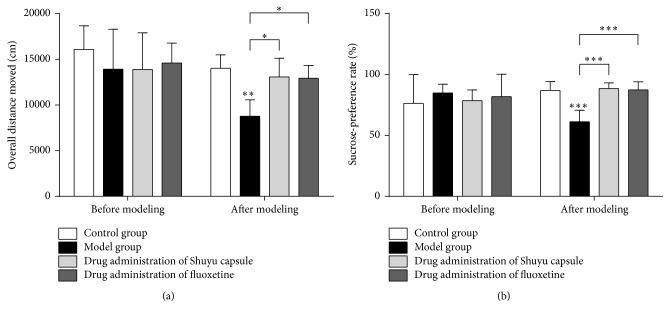
Behavioral assays. (a) Open-field test. (b) Sucrose-preference test. For all assays, testing was performed both before model building and after model building. Moreover, the following groups were analyzed: (1) the control/normal group (white), (2) model group (black), (3) Shuyu capsule group (gray), and (4) positive-control fluoxetine group (charcoal gray). The statistical analysis for the behavior assays was performed by one-way ANOVA (*n* = 18, ^*∗*^ *P* < 0.05, ^*∗∗*^ *P* < 0.01, and ^*∗∗∗*^ *P* < 0.001).

The overall path of movement in an open-field test is mainly used to illustrate animal locomotor activity (Figure 1(a)). Compared with rats in the normal control group, the overall path significance of rats in the model group was reduced (*P* < 0.01); compared with the model group, the overall path scores of rats given Shuyu capsule and fluoxetine both increased significantly (*P* < 0.05).

The sucrose-preference level of rodents is generally regarded as an expression of the reward response, and depressive animals commonly show a lowered sucrose preference [29]. Compared with the normal group, the sucrose-preference level of the model rats declined significantly (*P* < 0.001), demonstrating the occurrence of typical depressive mood changes in model rats and, hence, demonstrating that the model was successfully constructed (Figure 1(b)). Administration of the Shuyu capsule as well as the positive control (fluoxetine) mitigated the reduction in sucrose intake (Figure 1(b)) in the respective model animal groups (*P* < 0.001); there was no significant difference between the two treated groups.

### 3.2. Shuyu Capsules Effectively Reduce the Expression of 5-HT_3A_R and 5-HT_3B_R in Cerebral Regions in a Rat PMS Model

**Figure 2:**
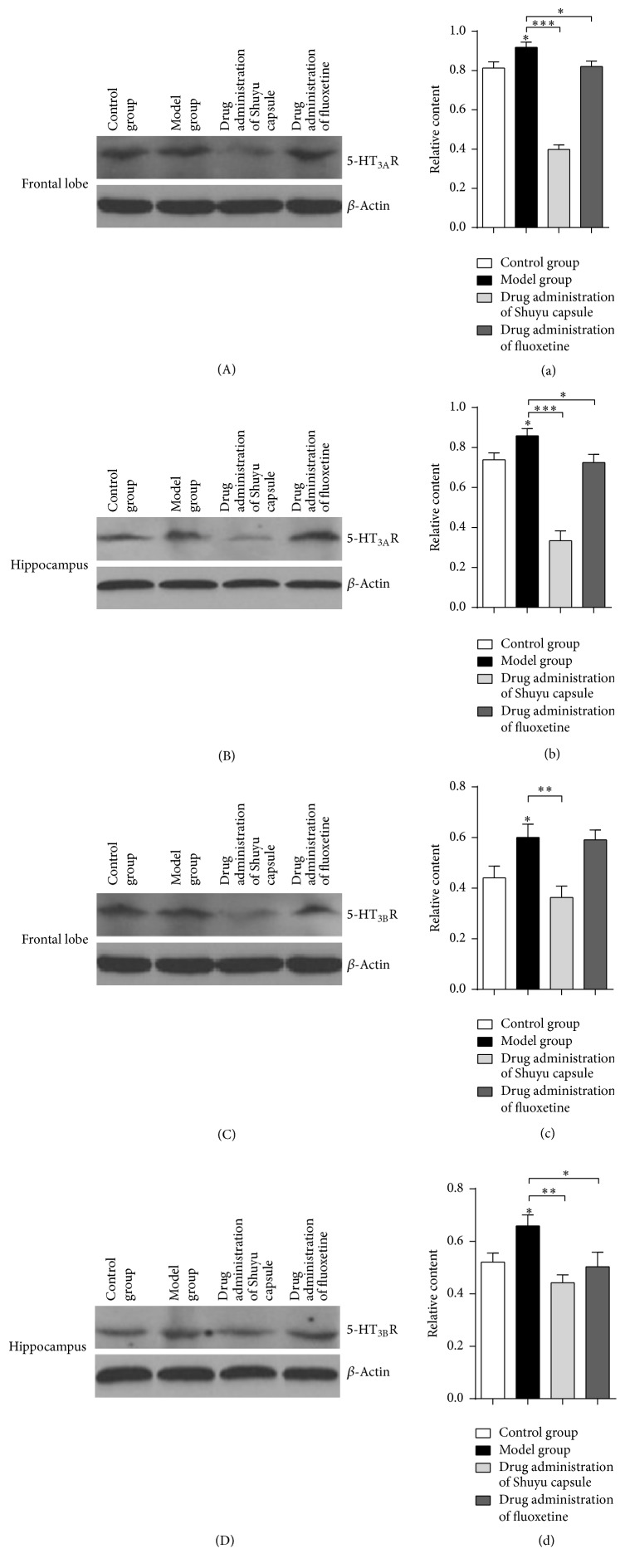
Western blot and analysis of tissue samples. Western blots of frontal lobe samples (A, C) and hippocampus samples (B, D) from each group, including the control/normal group, model group, Shuyu capsule group, and positive-control fluoxetine group. 5-HT_3A_R antibody was used (A, B) to define 5-HT_3A_R protein levels among the groups, while 5-HT_3B_R antibody was used (C, D) to define 5-HT_3B_R protein levels. A quantitative analysis (*n* = 6) of western blot results (a–d) was performed (^*∗*^ *P* < 0.05, ^*∗∗*^ *P* < 0.01, and ^*∗∗∗*^ *P* < 0.001).

Western blotting was used to detect the expression of 5-HT_3A_R and 5-HT_3B_R in different cerebral regions in the model rats. Regions in the frontal lobes and hippocampi revealed similarly increased expressions of 5-HT_3A_R in rats (Figures 2(A), 2(a), 2(B), and 2(b)); the expression level of 5-HT_3A_R in the model rats given the Shuyu capsule was significantly decreased as compared to the model group (Figures 2(A), 2(a), 2(B), and 2(b)). Animals in the positive-control group, which were given fluoxetine, showed a 5-HT_3A_R expression level that had recovered to a level similar to that of the normal group (Figures 2(A), 2(a), 2(B), and 2(b)). The expression of 5-HT_3B_R was very similar: the expression of 5-HT_3B_R was increased in model rats (Figures 2(C), 2(c), 2(D), and 2(d)), while it decreased significantly in rats given the Shuyu capsule (Figures 2(C), 2(c), 2(D), and 2(d)). However, the expression level of 5-HT_3B_R in the positive-control group given fluoxetine was restored to that of the normal group in the hippocampal area; yet, the expression of 5-HT_3B_R was not effectively lowered in the frontal region (Figures 2(C), 2(c), 2(D), and 2(d)).

**Figure 3:**
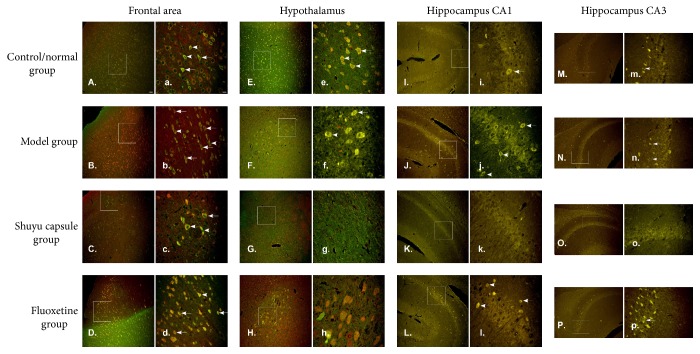
5-HT_3A_R- and 5-HT_3B_R-positive cell staining. 5-HT_3A_R- and 5-HT_3B_R-positive cells from different cerebral areas were stained, including the frontal lobe (A–D, a–d), hypothalamus (E–H, e–h), hippocampus CA1 (I–L, i–l), and hippocampus CA3 (M–P, m–p). The following samples were stained with both 5-HT_3A_R and 5-HT_3B_R antibodies: the control/normal group (A, E, I, M, a, e, I, and m), model group (B, F, J, N, b, f, j, and n), Shuyu capsule group (C, G, K, O, c, g, k, and o), and positive-control fluoxetine group (D, H, L, P, d, h, l, and p). 5-HT_3A_R- and 5-HT_3B_R-positive cells were defined by the merged signal (yellow). Scale bar (A–P): 100 *μ*m; scale bar (a–p): 25 *μ*m.

The expression of 5-HT_3A_R and 5-HT_3B_R in different cerebral areas showed similar increasing and decreasing trends, which raised the possibility of colocalizing these proteins. Fluorescence immunohistochemistry was used to determine the distributions of these proteins in different cerebral areas and their colocalization. Cells that coexpressed 5-HT_3A_R and 5-HT_3B_R in cerebral areas of different groups of rats showed yellow fluorescence (indicated by an arrow in Figure 3), with no apparent difference in the distribution mode. Positive cells were distributed in the frontal lobe area and hypothalamus and were present in the cell membranes of the CA1 and CA3 areas of the hippocampus, with most cells having dot and cone shapes (Figures 3(A–P) and 3(a–p)). The density of cells positive for colocalized 5-HT_3A_R and 5-HT_3B_R signals in the different groups paralleled the western blotting results. For example, in the frontal lobe (Figures 3(A–D) and 3(a–d)) and in the hypothalamus (Figures 3(E–H) and 3(e–h)), as well as in the CA1 area of the hippocampus (Figures 3(I–L) and 3(i–l)), the density of 5-HT_3A_R- and 5-HT_3B_R-positive cells in the model group far outnumbered that in the normal control group, and the density of 5-HT_3A_R- and 5-HT_3B_R-positive cells in rats given the Shuyu capsule was significantly decreased as compared with that in the model group. On the other hand, in the CA3 area of the hippocampus (Figures 3(M–P) and 3(m–p)), there were no marked differences in the 5-HT_3A_R- and 5-HT_3B_R-positive cell density between the model group and the normal control group, but in rats given the Shuyu capsule, the density of these cells was significantly decreased as compared with the model group.

### 3.3. Shuyu Capsules Effectively Reduce the Expression of 5-HT_3A_R and 5-HT_3B_R in Rat Hippocampal Neurons

**Figure 4:**
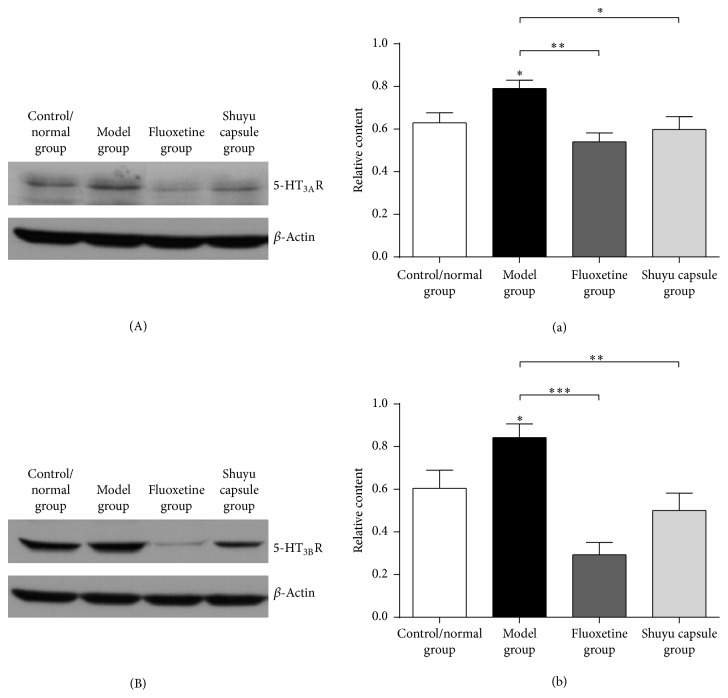
Western blot and primary hippocampal neuron analysis. The following drug serums were extracted and employed to treat primary hippocampal neurons: control/normal group, model group, positive-control fluoxetine group, and Shuyu capsule group. Both antibodies to 5-HT_3A_R (A) and 5-HT_3B_R (B) were used to test protein levels in hippocampal neurons among the groups. A quantitative analysis (*n* = 6) of the western blot results (a, b) was performed (^*∗*^ *P* < 0.05, ^*∗∗*^ *P* < 0.01, and ^*∗∗∗*^ *P* < 0.001).

We treated primary hippocampal neuron cultures with a drug serum that was extracted from the rats in the different groups, including the normal control group, the model group, the Shuyu capsule administration group, and the fluoxetine administration group. Compared with the normal group, 5-HT_3A_R protein expression was significantly increased in the hippocampal neurons in the model group (*P* < 0.05). Compared with the model group, 5-HT_3A_R protein expression in the fluoxetine administration group (*P* < 0.01) and Shuyu capsule administration group (*P* < 0.05) was significantly decreased (Figures 4(A) and 4(a)). Moreover, there were no marked differences in 5-HT_3A_R protein expression between these two groups.

Furthermore, compared with the normal group (*P* < 0.05), 5-HT_3B_R protein expression in hippocampal neurons increased, but in the fluoxetine- (*P* < 0.001) and Shuyu capsule-treated groups (*P* < 0.01), 5-HT_3B_R expression was decreased compared with the model group (Figures 4(B) and 4(b)). There were no marked differences in 5-HT_3B_R protein expression between these two groups.

### 3.4. Shuyu Capsule Drug Serum Can Effectively Reduce the Current Density of 5-HT_3_ Receptors in Hippocampal Neurons

**Figure 5:**
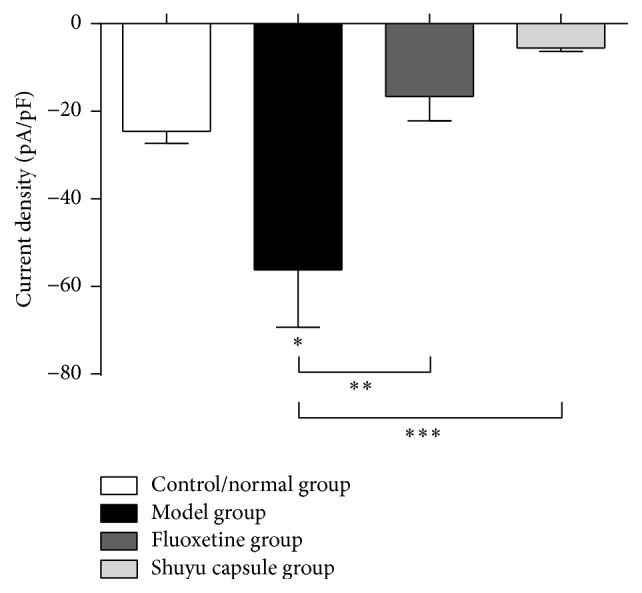
Whole-cell patch clamp analysis of primary hippocampal neurons. In this assay, 5-HT_3_R currents were recorded and analyzed for the following groups: the control/normal group (white), model group (black), positive-control fluoxetine group (charcoal gray), and Shuyu capsule group (gray). *n* = 6, ^*∗*^ *P* < 0.05, ^*∗∗*^ *P* < 0.01, and ^*∗∗∗*^ *P* < 0.001.

A drug serum containing the Shuyu capsule effectively reduced 5-HT_3_ receptor current density in hippocampal neurons (Figure 5). Compared with the normal group, the 5-HT_3_R current density of hippocampal neurons incubated with a serum was increased in the model group (*P* < 0.05). Compared with the model group, the 5-HT_3_R current density of hippocampal neurons incubated in the cells treated with Shuyu capsule-containing drug serum was significantly decreased (*P* < 0.001). Similar results were observed in the group given a fluoxetine-containing drug serum (*P* < 0.01).

## 4. Discussion

We had tested and verified face validity, construct validity, and predictive validity of PMS rat model in our previous studies [30–32]. In this work, we revealed that postmenstrual symptoms occurred in premenstrual phase and disappeared in postmenstrual symptoms in rat model. To be specific, rats showed the symptoms at the nonacceptance period (premenstruum) in estrous cycle and exhibited symptom relief or disappearance at the acceptance period (postmenstrual). Other groups verified PMS or premenstrual dysphoric disorder models with similar strategies and methods [33–35]. In current study, we selected healthy female Wistar rats through the open-field test and vaginal smear screening and created a PMS depression rat model by means of constraint [36] and evaluated these model animals using the open-field test and the sucrose-preference test. The significance of the overall path in model rats as well as their sucrose-preference level decreased, and some core depression symptoms, including listlessness, depression, and indifference, during the PMS period were well modeled. Through macroscopic behavior analysis, it was observed that rats in the model group were inactive and spiritless, had dull eyes, huddled together for sleep, were insensitive to external stimulation, were slow to respond, and slow to act. Combining the results of the open-field test and the sucrose-preference test, it was judged that model generation was successful.

In our previous study, a total of 73 compounds were identified in the Shuyu capsule [37]. In that study, rats were treated with the Shuyu capsule and their serum was analyzed. Thirteen novel compounds and 49 metabolites were found in rat serum; 14 metabolites were confirmed as novel metabolites of the Shuyu capsule. In the present study, we further investigated how the Shuyu capsule functioned in cerebral regions to begin to elucidate its mechanism of action.

Another study has shown that hormones, including estradiol and progesterone, can partially regulate the functions of the 5-HT receptor, including 5-HT_3_R, which is related to anxiety and depression [38]. Both estradiol and progesterone can function as noncompetitive antagonists of 5-HT_3_R [39] and reduce the expression of the corresponding mRNA in rats under stressful conditions [40]. The antidepressant and anxiolytic functions of estrogen have been widely recognized, and the underlying mechanism involves modulating the production, activity, and postsynaptic effect of various neurotransmitters, such as 5-HT and GABA, through the estrogen receptors (ERs) [41]. A previous study from our group [37] has shown that a high estrogen level in the hippocampus and increased expression and activity of ER*α* and ER*β* may play an important role in the pathogenesis of PMS depression [42]. The current study demonstrated the antidepressant effect of the Shuyu capsule in a rat model of PMS depression. The western blot results revealed that the Shuyu capsule can significantly reduce 5-HT_3A_R and 5-HT_3B_R protein expression in the frontal lobe and hippocampus, similar to the antidepressant and anxiolytic effects of estrogen, which is accompanied by a decrease in the expression of 5-HT_3A_R and 5-HT_3B_R.

5-HT_3_R forms a nonselective cation channel after being activated, through which sodium, potassium, and calcium ions can pass. The typical antidepressant fluoxetine clearly inhibits the delayed-rectifier potassium current on cerebellar granule neurons, as well as the transient outward potassium current on hippocampal neurons. When used as depression treatment, fluoxetine can also increase cell excitability, by inhibiting the voltage-dependent delayed-rectifier potassium current, hence increasing the antidepressant effect [43, 44]. Some researchers have proposed that a selective 5-HT-reuptake inhibitor, such as fluoxetine, exerts its antidepressant effect by blocking the potassium channel in the 5-HT_3_R coupling [45]. The results of this present* in vitro* study showed that the expression of 5-HT_3A_R and 5-HT_3B_R proteins in hippocampal neurons incubated with PMS depression model rat sera was significantly increased and that the PMS depression model rat serum activated the 5-HT_3_R channel current. This was consistent with the earlier findings that opening of a potassium channel led to the development of depressive symptoms [46–48]. The expression of 5-HT_3A_R and 5-HT_3B_R in hippocampal neurons incubated with drug sera from rats in the Shuyu capsule administration group was decreased; moreover, the channel current was also hindered. This is probably because the potassium channel current in the 5-HT_3A_R and 5-HT_3B_R coupling was hindered.

## Supplementary Material

Graphical abstract

## Abbreviations

5-HT_3_: 5-Hydroxytryptamine 3 receptor
ANOVA: Analysis of variance
DMSO: Dimethyl sulfoxide
HRP: Horseradish peroxidase
NBG medium: Nutrient broth-glucose medium
PBG: Phenylbiguanide
PMS: Premenstrual syndrome
TCM: Traditional Chinese medicine
PBS: Phosphate-buffered saline.
