# Supplementary figures and images for: Shuyu Capsules Relieve Premenstrual Syndrome Depression by Reducing 5-HT3AR and 5-HT3BR Expression in the Rat Brain
Source: Neural Plast. 2016 Sep 20;2016:7950781. doi: 10.1155/2016/7950781 (PMC5048033; doi:10.1155/2016/7950781)

# 5-HT<sub>3A/3BR</sub>

T

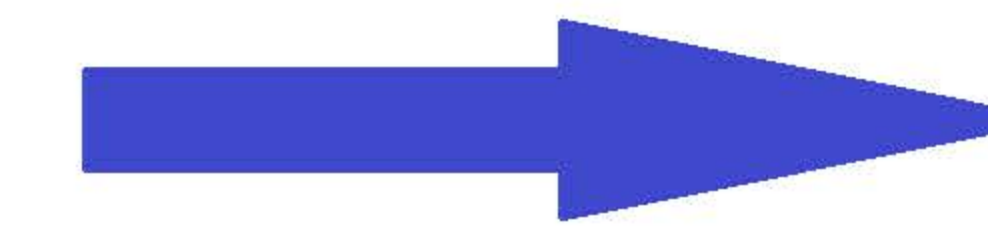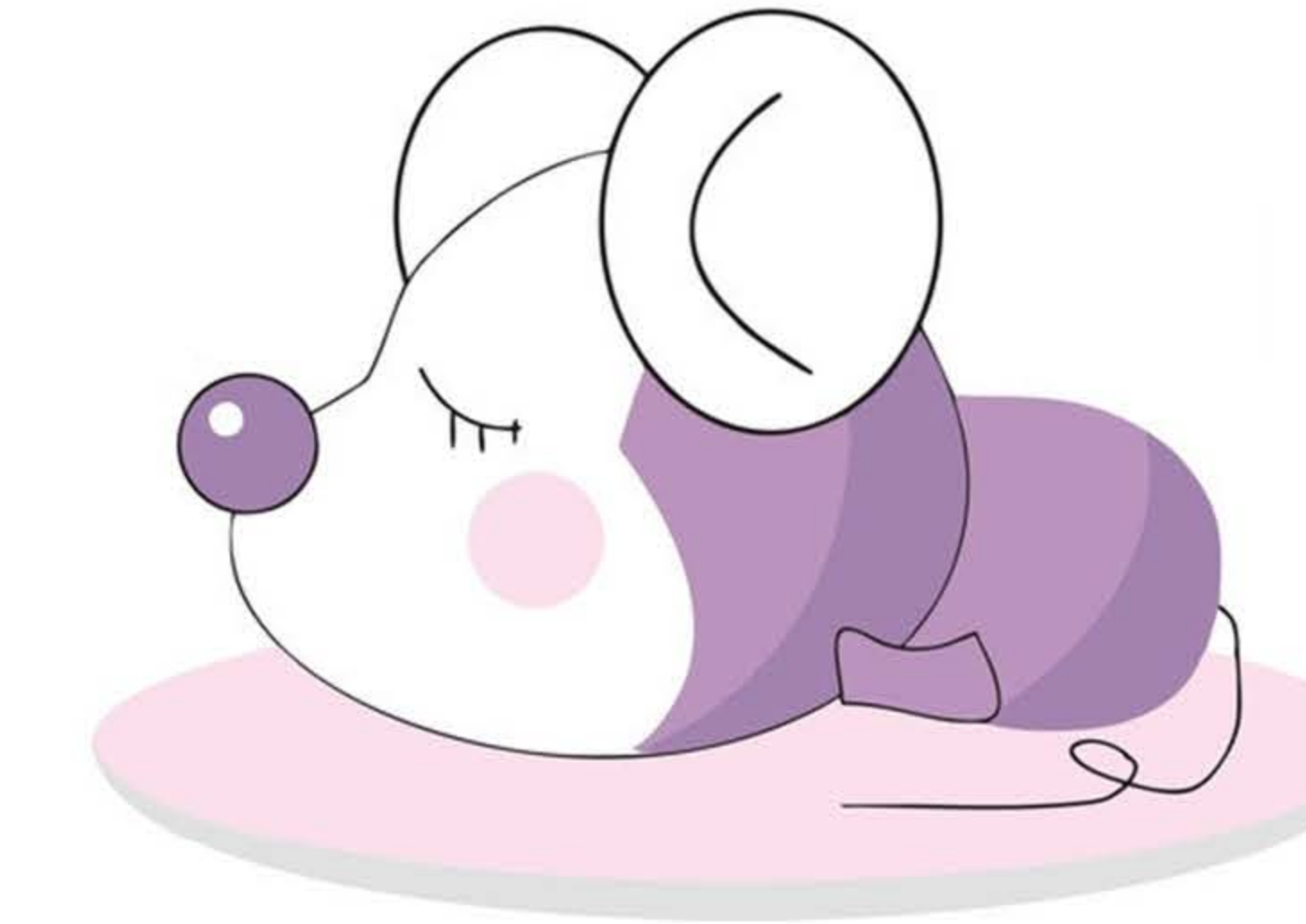

## Relieving PMS Depression

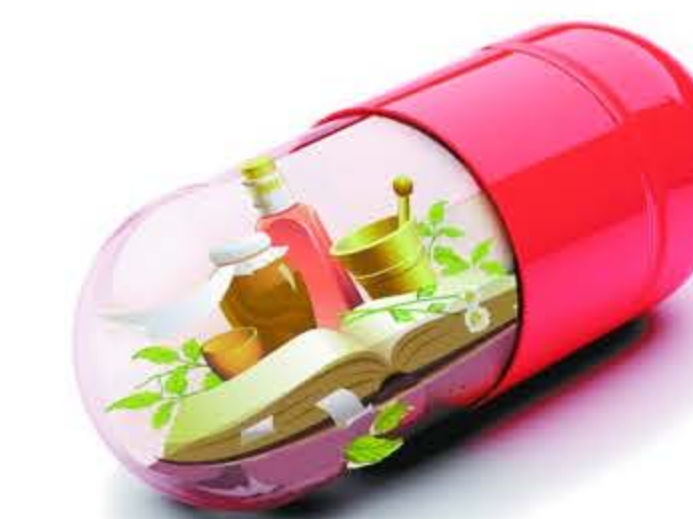

### Shuyu Capsule

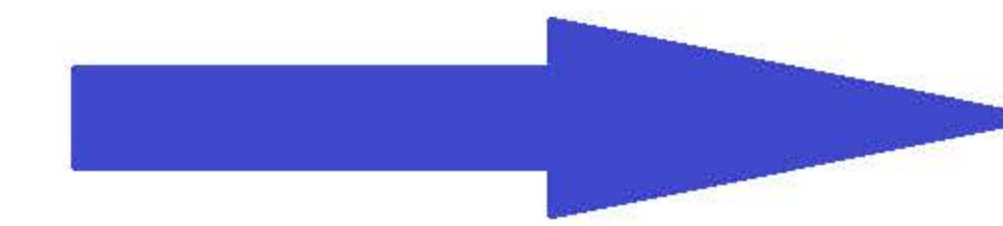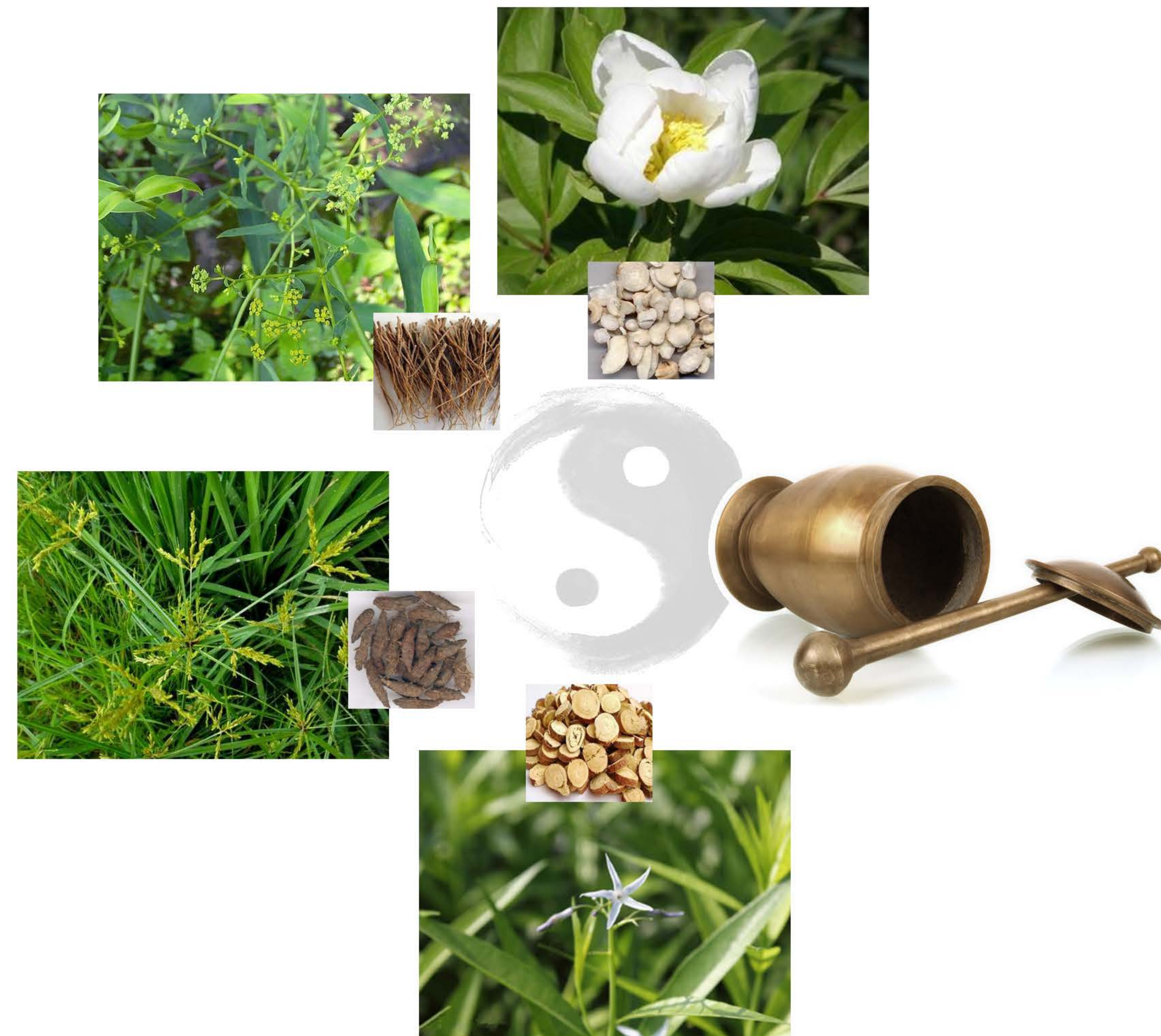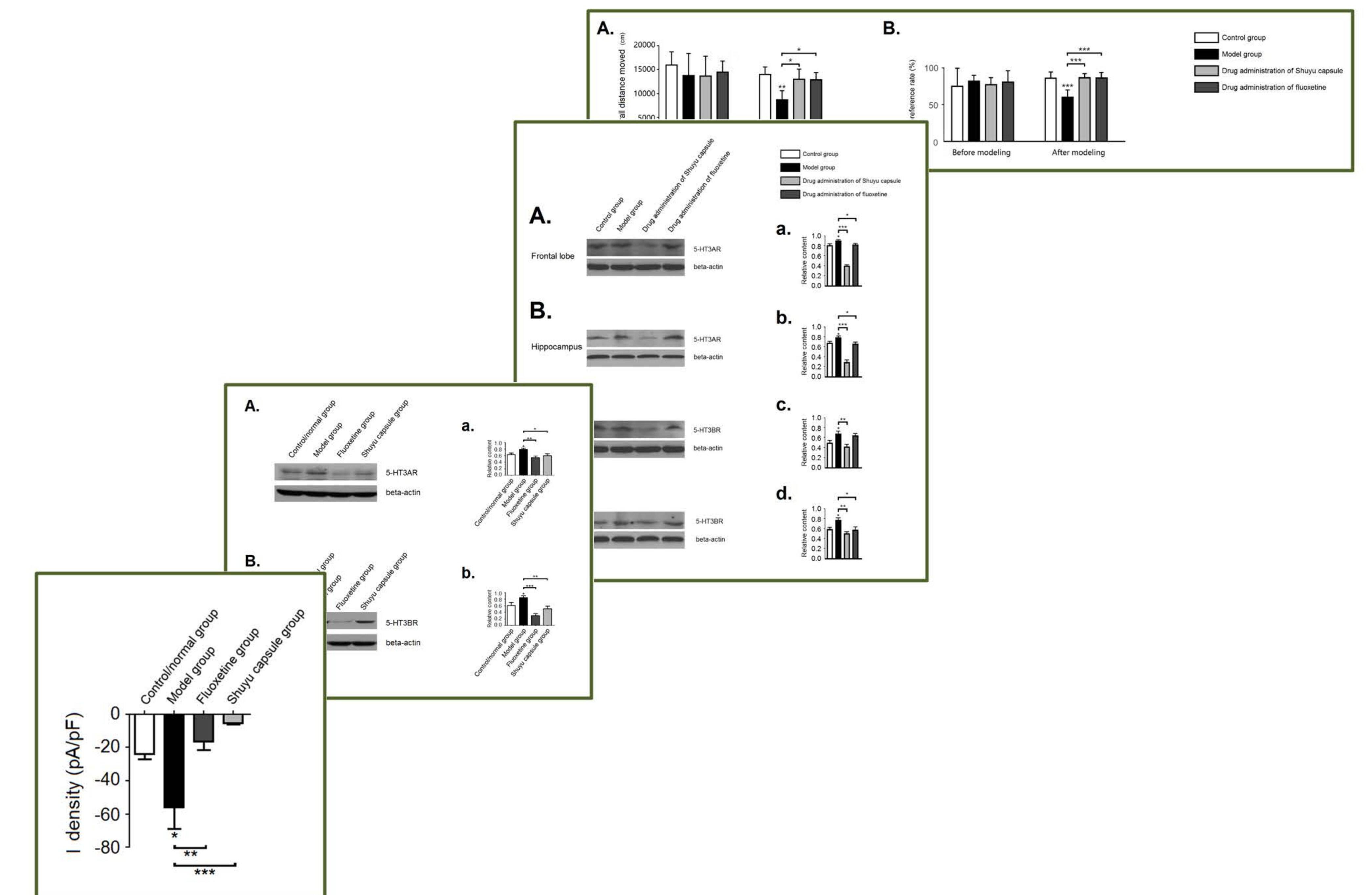

Supplement: Supplementary file 1 — Graphical abstract [file 7950781.f1.pdf]
